# Reducing short-acting beta-agonist use in asthma: Impact of national incentives on prescribing practices in England and the findings from SENTINEL Plus early adopter sites

**DOI:** 10.1038/s41533-024-00363-0

**Published:** 2024-04-29

**Authors:** M. G. Crooks, H. Cummings, A. H. Morice, D. Sykes, S. Brooks, A. Jackson, Y. Xu

**Affiliations:** 1 Hull York Medical School, University of Hull, Hull, UK; 2 Hull University Teaching Hospitals NHS Trust, Hull, UK; 3 Medical Affairs, AstraZeneca, London, UK

**Keywords:** Health policy, Outcomes research

## Abstract

Short-acting beta-agonist (SABA) over-use in asthma is harmful for patients and the environment. The Investment and Impact Fund (IIF) 2022/2023 financially rewarded English primary care networks that achieved specific targets, including reducing SABA over-use (RESP-02) and lowering the mean carbon footprint per salbutamol inhaler prescribed (ES-02). SENTINEL Plus is a co-designed quality improvement package that aims to improve asthma outcomes and reduce asthma’s environmental impact by addressing SABA over-use. We investigated the impact of (i) the IIF incentives and (ii) SENTINEL Plus implementation on asthma prescribing. Using Openprescribing.net data, we demonstrate that IIF 2022-2023 had no significant impact on the total number of SABA prescribed in England (25,927,252 during 12-months pre- and 25,885,213 12-months post-IIF; 0.16% decrease; p=NS), but lower carbon footprint SABA inhaler use increased (Salamol™ prescribing increased from 5.1% to 19% of SABA prescriptions, *p* < 0.01). In contrast, SENTINEL Plus sites significantly reduced SABA prescribing post-implementation (5.43% decrease, *p* < 0.05).

## Introduction

Short-acting beta-agonist (SABA) over-use in asthma (≥3 SABA inhalers per year) is associated with increased risk of exacerbation^1^ and death^2,3^. Despite this, SABA inhalers are the most commonly prescribed inhaler in England, 94% of which are greenhouse gas-containing pressurised metered dose inhalers (pMDI)^4^.

The National Health Service (NHS) in England recognises the patient and environmental harms associated with SABA over-use in asthma and, until recently, offered financial incentives to try to address this. The Investment and Impact Fund (IIF) (2022/2023)^5^ rewarded primary care networks (PCNs) that achieved key targets including: RESP-02, which aimed to reduce the proportion of registered asthma patients receiving ≥6 SABA inhalers per year; and ES-02, which incentivised reduction of the mean carbon emissions per salbutamol inhaler prescribed. While reducing SABA over-use has both patient and environmental benefit, prescribing lower carbon SABA carried a greater IIF incentive but was purely environmentally focussed. This led some to undertake inhaler switch programmes, in which higher-carbon SABA inhalers were replaced with lower-carbon alternatives.

PrescQIPP, a not-for-profit community interest company that aims to improve medicines-related care for NHS patients, has published resources relating to inhaler carbon emissions. They endorse switching Ventolin Evohaler™, a commonly issued SABA inhaler in England, to Salamol™ pMDI to achieve environmental and cost savings^6^. The National Institute of Health and Care Excellence (NICE) also produce a decision aid to help inform inhaler choice with regard to carbon footprint^7^. PrescQIPP state that any switch should be individually tailored and involve shared decision-making; however, contrary to this, switch programmes are sometimes undertaken at scale and without patient review^8,9^.

SENTINEL Plus is a quality improvement package that aims to improve outcomes for asthma patients and reduce the environmental impact of asthma treatment by identifying and addressing SABA over-use. SENTINEL Plus utilises a co-designed intervention, developed during the SENTINEL Project^10^, with 5 components: (i) health care professional education, (ii) patient support and education, (iii) targeted asthma reviews for patients over-using SABA, iv) adherence to prescribing ‘gold standards’; and (v) data monitoring and feedback. SENTINEL Plus supports guideline-recommended asthma care delivery, including SABA-free Maintenance and Reliever Therapy (MART) where appropriate. This framework has demonstrated promising results, with data from the pilot PCN showing reduced SABA prescribing, leading to attainment of RESP-02, and improved patient outcomes^11^.

In this article, we analyse SABA and ICS prescribing in England during the 12-months before and after introduction of the 2022/2023 IIF incentives and explore the impact of SENTINEL Plus implementation in early adopter sites in England. To explore the interaction between RESP-02 and ES-02 in practice, we compare the differential prescribing changes between SENTINEL Plus early adopter sites with and without evidence of having also undertaken a switch programme in favour of lower carbon SABA inhalers.

## Results

### SABA and ICS prescribing in England before and after IIF 2022/2023 introduction

Total SABA prescribing in England remained essentially unchanged in the 12-months following the introduction of IIF 2022/2023 incentives, reducing by 0.16%, from 25,927,252 to 25,885,213 (*p* = 0.9). Salamol™ prescribing increased significantly from 1,322,729 prescriptions in the 12-months before to 4,931,087 prescriptions the year after (*p* < 0.001), with Salamol™ accounting for 19% of all SABA prescriptions post-IIF, from 5% pre-IIF. ICS prescribing increased by 508,124 prescriptions across England, representing a 2% increase from the year before (*p* = 0.05). A summary of England prescribing data is presented in Table 1.

**Table 1:** SABA and ICS prescribing in primary care in England for the 12-months before (April 2021–March 2022) and after (April 2022-March 2023) the Impact and Investment Fund 2022-2023 was introduced, including incentives RESP-062 and ES-02.

Descriptor	All Primary Care Prescribing in England	
Total Prescribing	Pre-IIF April 2021–March 2022	Post-IIF April 2022–March 2023
**SABA -** ***n***	25,927,252	25,885,213
**ICS -** ***n***	26,375,509	26,883,633
**Proportion SABA (%)**	50%	49%
**SABA Prescribing in England**	**Pre-IIF** **April 2022–March 2023**	**Post-IIF** **April 2022–March 2023**
**All SABA –** ***n***	25,927,252	25,885,213
- **Salamol™ –** ***n*** ** - Ventolin Evohaler™**	1,322,729 5,528,836	4,931,087 4,414,140
**Salamol as a proportion of all SABA (%)**	5.1%	19%
**Change in Prescribing** **Apr 21–Mar 22 to Apr 22–Mar 23**	**All Primary Care Prescribing in England**	
**SABA**		
- ***n*** - **% change (post-IIF vs. pre-IIF)**	−42,039 −0.16%	
**-Salamol**		
- ***n*** - **% change (post-IIF vs. pre-IIF)**	+3,608,358^#^ +273%	
**-Ventolin Evohaler**		
- ***n*** - **% change (post-IIF vs. pre-IIF)**	−1,114,696^#^ −20%	
**ICS**		
- ***n*** ** - % change from prior 12-months**	+508,124** +1.93%	
**Proportion SABA (%)**	−0.52%	

Statistical analysis undertaken using paired t-tests to compare prescribing before and after RESP-02 and ES-02 introduction. **p = 0.05, #*p* ≤ 0.01.

### SABA and ICS prescribing in SENTINEL Plus early adopter sites in England: before and after implementation

A summary of prescribing data before and after SENTINEL Plus implementation is presented in Table 2.

**Table 2:** SABA and ICS prescribing post-SENTINEL Plus implementation compared with predicted prescribing based on pre-SENTINEL Plus prescribing levels.

Descriptor	Total population		Practices with no additional SABA switch programme		Practices with additional SABA switch programme	
Number of practices—*n*	25		16		9	
Population size—*n*	456,135		267,451		188,684	
Asthma patients—*n*	29,349		17,412		10,937	

Total prescribing	Predicted prescribing (based on pre-SENTINEL baseline data)	Observed prescribing post-SENTINEL	Predicted prescribing (based on pre-SENTINEL baseline data)	Observed prescribing post-SENTINEL	Predicted prescribing (based on pre-SENTINEL baseline data)	Observed prescribing post-SENTINEL
SABA—*n*	192,954	182,473	129,376	120,064	63,578	62,407
- of which Salamol—*n*	4817	17,205	3706	5731	1111	11,474
ICS—*n*	204,504	210,482	132,152	135,948	72,352	74,534
Proportion SABA (%)	49%	46%	49%	47%	47%	46%

Change in prescribing post-SENTINEL Plus	Total population		Practices with no SABA switch programme		Practices with a SABA switch programme	
*SABA*						
All SABA—*n*	−10,481*		−9312**		−1171	
% change from prev 12 m	−5.43%		−7.2%		−1.84%	
- Salamol—*n*	+12,388*		+2025^#^		+10,363*	
% change from prev 12 m	+257%		+55%		+933%	
*ICS*						
*n*	+5978^#^		+3796^#^		+2182^#^	
% change from prev 12 m	+2.92%		+2.87%		+3.02%	
Proportion SABA (%)	−3%		−2%		−1%	

Change in prescribing post-SENTINEL Plus per practice per month						
SABA—mean [95% CI]	−30.8 [−55.6 to −6.1]		−43.3 [−79.9 to −6.8]		−8.6 [−34.3 to 16.1]	
ICS—mean [95% CI]	+19.0 [9.7 to 28.4]		+18.3 [4.5 to 32.0]		+20.4 [7.4 to 33.4]	

Statistical analysis undertaken using paired t-tests to compare prescribing before and after SENTINEL Plus implementation: *p < 0.05, **p = 0.05, ^#^*p* ≤ 0.01.

**Fig. 1:**
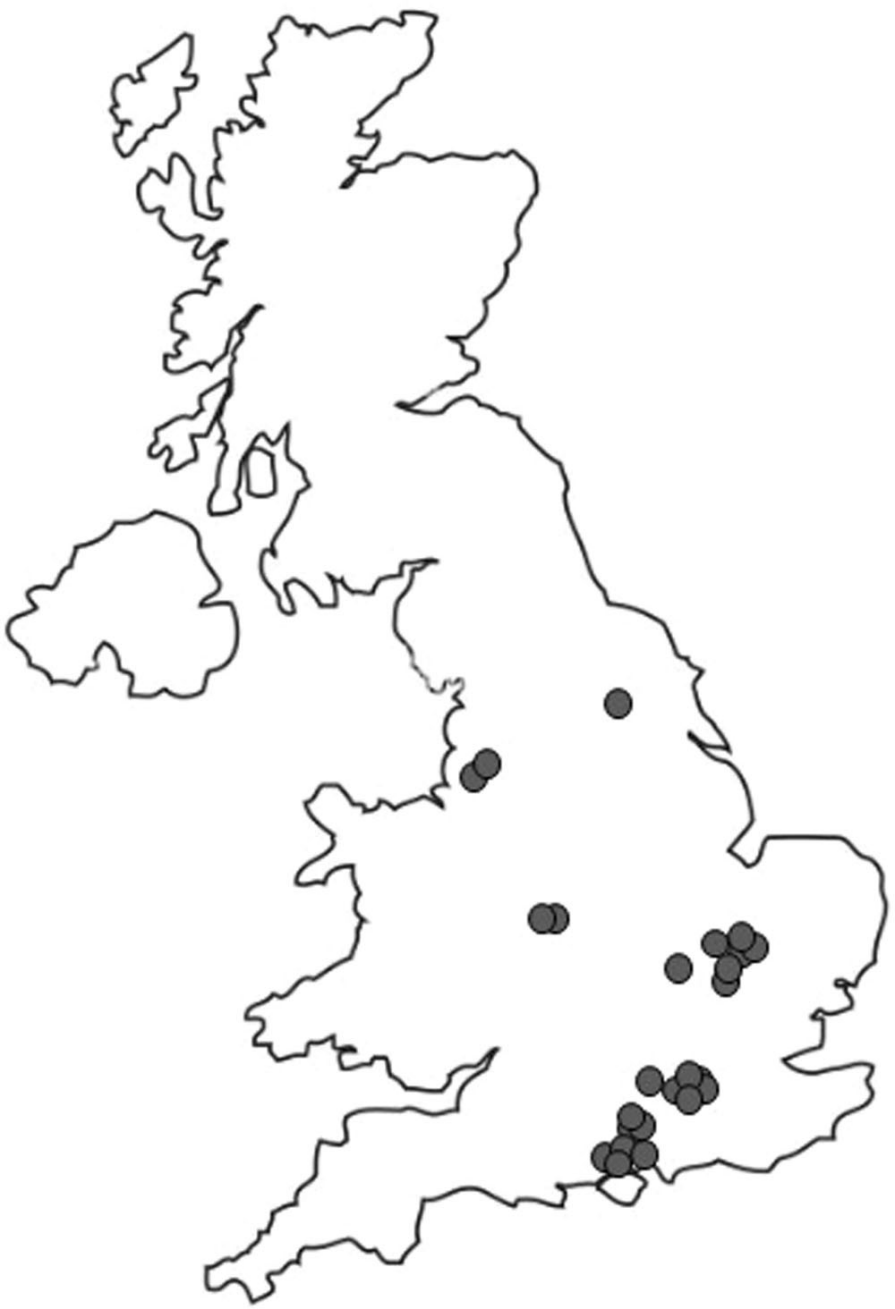
Location of early SENTINEL Plus sites.

The first 25 SENTINEL Plus practices in England were geographically spread with clusters in Cambridgeshire and Hampshire (Fig. 1). The median (range) practice list size was 12,777 (5,918 to 59,763) people with median 828 (281 to 3741) registered asthma patients. The mean follow-up post-SENTINEL Plus implementation was 11.2 months (range 8 to 16 months). A total of 281 months of follow-up prescribing data were available for analysis.

10,481 fewer SABA inhalers were prescribed post-SENTINEL Plus implementation than expected if prescribing had remained unchanged from pre-SENTINEL Plus levels (*p* < 0.05). This represents a 5% reduction in all SABA prescribing and a mean change of −30.83 (95% CI −55.59 to −6.07) SABA prescriptions per practice per month. ICS prescribing increased following SENTINEL Plus with 5,978 more ICS-containing inhalers prescribed across early adopter sites compared with pre-SENTINEL Plus levels (*p* < 0.001).

**Fig. 2:**
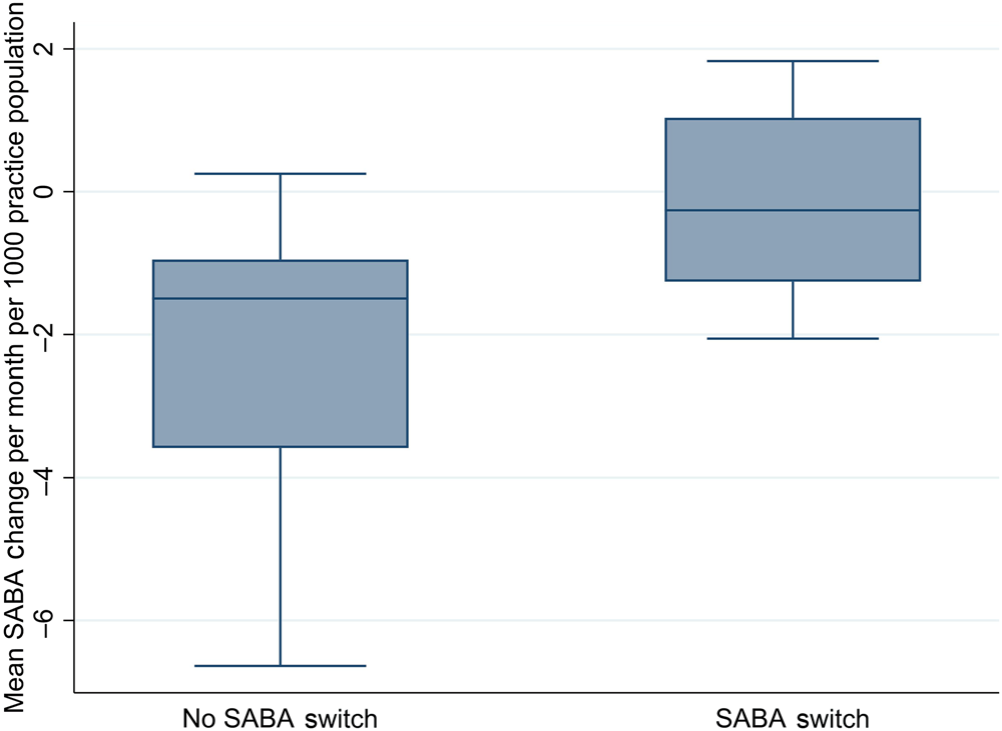
Change in SABA prescribing in SENTINEL Plus early adopter sites. Box Plot depicting change in SABA prescribing per month per 1,000 practice population for practices with evidence of undertaking a SABA switch programme within 12-months of SENTINEL Plus compared with those not undertaking a SABA switch programme (center line, median; box limits, upper and lower quartiles; whiskers, 1.5x interquartile range. Unpaired, two-sided t-test: p < 0.01).

Following the review of individual practice SABA prescribing, 9 out of the 25 SENTINEL Plus early adopter sites were identified as having also undertaken a SABA switch programme to increase Salamol™ use. Accounting for differences in follow-up duration and practice list size, practices not undertaking an additional SABA switch programme had a significantly greater reduction in SABA prescribing compared with practices that undertook a switch programme within 12-months of SENTINEL Plus implementation (Fig. 2, *p* < 0.01). The increase in ICS prescribing was similar across SENTINEL Plus early adopter sites, irrespective of whether they engaged in an additional SABA switch programme or not (Table 2).

## Discussion

Implementation of IIF 2022/2023 incentives in England did not lead to any meaningful reduction in overall SABA prescribing but was associated with an almost 4-fold increase in Salamol™ use, reflecting a move away from Ventolin Evohaler™ and generic salbutamol prescribing (a proportion of which is dispensed as Ventolin Evohaler™). While this will have environmental benefits, given the lower carbon impact of Salamol™, the lack of change in SABA prescribing as a whole shows that SABA over-use was not tackled at scale. This suggests that incentive schemes, such as the IIF, maybe sufficient to bring about apparently simple changes in prescribing practice (between brand prescription changes), albeit with potential to promote scaled prescription switching. However, the lack of impact on overall SABA prescribing suggests that incentives alone may not be sufficient to achieve more complex changes in care, such as addressing SABA over-use.

In contrast, SENTINEL Plus early adopter sites achieved a significant reduction in SABA prescribing in the period after SENTINEL Plus implementation, confirming its ability to support clinical practice change across diverse primary care sites in England. Electronic health record data from >2500 asthma patients in the SENTINEL Project pilot PCN demonstrated similar prescribing changes and a reduction in asthma exacerbations post-implementation^11^, supporting an assumption that the observed prescribing changes will translate into patient benefit.

Significantly lower reductions in SABA prescribing were observed among early adopter practices that appeared to have also engaged in a SABA switch programme. Many factors may influence this observation, and it is not possible to conclude cause and effect based on the presented data. However, it is important to consider whether incentives to promote lower carbon SABA inhaler use may have adversely affected efforts to tackle SABA over-use as a whole. Indeed, addressing SABA over-use in asthma is challenging due to the complex and often numerous contributory patient, clinician, and health system factors^12,13^. Changing both clinician and patient behaviour is vital and clear messaging about the harms of SABA over-use in asthma is essential. This may be undermined by concurrent efforts to promote an alternative, less environmentally harmful SABA. Something that will continue to be relevant with the advent of new propellant gasses that will lower the environmental impact further^14^.

The nature and weighting of IIF Target ES-02^5^ also meant that a practice that significantly reduced their overall SABA prescribing but did not lower the mean carbon footprint per SABA, would have received less funding through the IIF than a practice that increased overall SABA prescribing, but did so using lower carbon inhalers. This is despite the former scenario having greater patient and environmental benefit.

For 2023/2024, the IIF has changed to focus on healthcare access, with loss of specific incentives to transform asthma care. This is leading local healthcare commissioners to consider introducing bespoke prescribing targets for their regions to continue to drive improvements aligned to the NHS long-term plan. It is therefore timely to consider whether greater synergy between patient and environmentally focussed incentives could be achieved by i) recognising the carbon savings from reduced overall SABA prescribing in asthma and ii) balancing financial incentives to prioritise patient benefit. While incentives alone may not be sufficient to overcome complex healthcare challenges, they have an important role in supporting practice change and should be considered alongside quality improvement packages to maximise impact. We would therefore strongly support reintroduction of RESP-02 but with aligned implementation support using a quality improvement package such as SENTINEL Plus.

When considering the implications of our findings, it is important to acknowledge the limitations of this study. Openprescribing.net data includes all primary care prescriptions and therefore captures prescribing for other respiratory conditions, such as COPD, in addition to asthma. This is less of a limitation when considering measures that span conditions, such as ES-02, but would be expected to limit the ability to detect change among specific sub-groups within the whole population, such as adult asthma patients that over-use SABA, the focus of SENTINEL Plus. Despite this, we observed a significant reduction in SABA and increase in ICS prescribing among early adopter sites, indicating the ability to detect changes in asthma prescribing using population-level data. Another limitation is the absence of matched controls for our SENTINEL Plus early adopter sites, raising the potential that factors other than the intervention may have impacted prescribing. However, analysis of national prescribing data did not reveal any significant change in SABA prescribing at a population level, increasing the likelihood that the change in prescribing observed post-SENTINEL Plus implementation is a consequence of the intervention, rather than a wider trend. Findings from the SENTINEL Project pilot site also support the interventions effectiveness^11^.

When complete, the full SENTINEL Project effectiveness evaluation will provide definitive evidence about the impact of the SENTINEL intervention on prescribing, clinical outcomes and the environmental impact of asthma care. An associated implementation evaluation will provide insights into barriers and facilitators to successful implementation. However, while this is awaited, we present promising data from early SENTINEL Plus adopting practices and suggest that concurrent programmes to switch higher carbon to lower carbon SABA inhalers at scale have potential to negatively impact efforts to overcome SABA over-use as a whole and should be approached with care.

In conclusion, total SABA prescribing in England remained unchanged during the 12-months following IIF 2022/2023 introduction but there was a significant shift to use of lower carbon impact SABA inhalers. SENTINEL Plus implementation within primary care practices in England was associated with a significant reduction in total SABA prescribing; but, practices that undertook additional SABA switch programmes were less likely to achieve these overall reductions. It is therefore important for policy makers to develop incentives that act synergistically to achieve the desired practice change. To tackle SABA over-use at scale, consideration should be given to aligning incentives with quality improvement interventions.

## Methods

### SABA and ICS prescribing in England

To evaluate changes in prescribing practice across the whole of England, aligned with introduction of IIF incentives RESP-02 and ES-02, prescribing data (OpenPrescribing.net, Bennett Institute for Applied Data Science, University of Oxford, 2023) were analysed for the months April 2021–March 2022 (Pre-IIF) and April 2022 – March 2023 (post-IIF). Data for SABA-use and use of ICS-containing inhalers were obtained from the Openprescribing.net SABA inhaler dashboard dataset. Salamol™ data were collected for the same period through a bespoke search for monthly Salamol™ prescribing [Salamol code: 0301011R0BI] in England during pre- and post-IIF periods. Given the equivalent carbon footprint of Salamol pMDI and Salamol Easi-breath (a breath-actuated inhaler)^6^, prescribing data for both devices are presented together. Prescribing data are presented descriptively and before and after comparisons are made using paired t-tests.

### SABA and ICS prescribing in SENTINEL plus early adopter sites

Prescribing data (Openprescribing.net) were analysed for the first 25 GP practices to implement SENTINEL Plus in England for the months July 2020 to October 2022 inclusive, to include at least 12-months pre-SENTINEL Plus prescribing data for each practice. The number of SABA and ICS inhalers prescribed post-SENTINEL implementation were compared with data from matched months during the 12-months pre-implementation to account for seasonal variation in asthma prescribing. The differences between predicted prescribing, based on the observed prescribing during the 12-months pre-implementation, and the observed post-SENTINEL implementation prescribing were calculated.

Early adopter practices that undertook a SABA switch programme in addition to SENTINEL Plus were identified by a doubling of Salamol™ prescribing, to comprise at least 25% of all SABA prescriptions during the studied period. Given the lack of existing criteria to define a SABA switch programme based on prescribing data, the thresholds were chosen following review of practice level prescribing data. The selected thresholds provided a clear differentiation between practices with no change in Salamol™ prescribing and/or a gradual change, which could conceivably have occurred through a process of patient review, and practices in which there was a rapid and sustained shift in prescribing, considered more likely to have occurred through a prescription switch programme, without clinical review. Further information is available in supplementary material.

Individual practice prescribing data were adjusted for list size and follow-up duration and statistical analyses undertaken using either paired (before and after comparisons) or unpaired t-tests (between group comparisons), using Stata SE 17 (StataCorp LLC, USA). The normality of data distribution was confirmed using Shapiro Wilk test.

### Supplementary information

Supplementary Material

Reporting Checklist

## Data Availability

All data included in this analysis and publicly available (OpenPrescribing.net, Bennett Institute for Applied Data Science, University of Oxford, 2023).
