# Author Correction: Reducing short-acting beta-agonist use in asthma: Impact of national incentives on prescribing practices in England and the findings from SENTINEL Plus early adopter sites

**DOI:** 10.1038/s41533-024-00376-9

**Published:** 2024-07-09

**Authors:** M. G. Crooks, H. Cummings, A. H. Morice, D. Sykes, S. Brooks, A. Jackson, Y. Xu

**Affiliations:** 1 Hull York Medical School, University of Hull, Hull, UK; 2 Hull University Teaching Hospitals NHS Trust, Hull, UK; 3 Medical Affairs, AstraZeneca, London, UK

**Keywords:** Health policy, Outcomes research

Correction to: *npj Primary Care Respiratory Medicine* **34**, 6 (2024) 10.1038/s41533-024-00363-0, published online 29 April 2024

In this article the Table 2 columns were incorrectly structured. The original article has been corrected.

