# Supplementary material for: Reducing short-acting beta-agonist use in asthma: Impact of national incentives on prescribing practices in England and the findings from SENTINEL Plus early adopter sites
Source: NPJ Prim Care Respir Med. 2024 Apr 29;34:6. doi: 10.1038/s41533-024-00363-0 (PMC11058200; doi:10.1038/s41533-024-00363-0)
Supplement: Supplementary file 1 — Supplementary Material [file 41533_2024_363_MOESM1_ESM.pdf]

Reducing short-acting beta-agonist use in asthma: the impact of national incentives on prescribing practices in England and the findings from SENTINEL Plus early adopter sites.

Crooks MG[1,2], Morice AH[1,2], Cummings H[2], Sykes D[1,2], Brooks S[3], Jackson A[3], Xu Y[3].

Affiliations:

1. Hull York Medical School, University of Hull, Hull, United Kingdom
2. Hull University Teaching Hospitals NHS Trust, Hull, United Kingdom
3. Medical Affairs, AstraZeneca, London, United Kingdom

Supplementary Material

1. Identification of practices likely to have engaged in a SABA switch programme

There are no existing criteria to define a SABA switch programme base on Openprescribing.net data. Therefore, individual practice level prescribing data were scrutinised in order to identify changes in prescribing practice that were considered by the researchers to be consistent with a scaled programme of SABA prescription switching. This was undertaken prior to analysis of SABA and ICS prescribing patterns.

Following review of the data, the following criteria were considered consistent with a switch programme:

- At least a doubling of Salamol™ prescribing as a proportion of all SABA inhalers
- Salamol™ prescribing increased to comprise at least 25% of all SABA prescriptions

An example of monthly Salamol™ prescribing for 2 practices identified as having undertaken a SABA switch programme and 2 practices identified as not having undertaken a SABA switch programme are presented below.

SABA switch programme

	Salamol™ as a proportion of all SABA prescribing											
	Month 1	Month 2	Month 3	Month 4	Month 5	Month 6	Month 7	Month 8	Month 9	Month 10	Month 11	Month 12
**Practice 2**	5%	7%	6%	8%	8%	7%	9%	11%	10%	41%	63%	63%
**Practice 6**	3%	5%	4%	5%	23%	60%	68%	63%	64%	61%	62%	64%

Supplementary Table 1. An example of Salamol™ prescribing over 12-months, as a proportion of all SABA prescribing, for 2 practices identified as likely having undertaken a scaled SABA switch programme. Month's identified as meeting criteria for a SABA switch programme having taken place are highlighted in red.

No SABA switch programme

	Salamol™ as a proportion of all SABA prescribing											
	Month 1	Month 2	Month 3	Month 4	Month 5	Month 6	Month 7	Month 8	Month 9	Month 10	Month 11	Month 12
**Practice 1**	3%	2%	2%	2%	3%	3%	3%	2%	3%	3%	3%	4%
**Practice 7**	7%	7%	6%	7%	6%	7%	8%	9%	10%	11%	10%	15%

Supplementary Table 2. An example of Salamol™ prescribing over 12-months, as a proportion of all SABA prescribing, for 2 practices identified as not having evidence of having undertaken a scaled SABA switch programme.
